# Supplementary material for: Metabolite Profiling of the Resurrection Grass Eragrostis nindensis During Desiccation and Recovery
Source: Plants (Basel). 2025 Feb 9;14(4):531. doi: 10.3390/plants14040531 (PMC11859761; doi:10.3390/plants14040531)
Supplement: Supplementary file 1 [file plants-14-00531-s001.zip › Supplementary tables and figures.pdf]

Supplementary data

Table S1: Shows differentially abundant metabolites in both senescent and non-senescent tissue during dehydration and rehydration. (P < 0.05)

Metabolite	f.value	p.value	Metabolite	f.value	p.value
2.3-Butanediol	19.24	1.29E-13	Glycerol 1-phosphate	4.48	0.00010077
Glyceryl-glycoside	18.938	1.69E-13	D-mannitol	4.448	0.00010832
Dehydroascorbic acid	16.43	1.88E-12	L-Phenylalanine	4.4382	0.00011075
xylitol	16.139	2.53E-12	L-lysine	4.3312	0.00014124
D-galactose	15.542	4.73E-12	Ribose	4.2337	0.00017658
Lactic acid	14.277	1.89E-11	L-Tryptophan	4.0432	0.0002747
Melibiose	13.948	2.76E-11	Sucrose	3.899	0.0003856
D-Arabinose	13.728	3.56E-11	L-threonine	3.8629	0.00042
L-leucine	13.577	4.25E-11	Maltitol	3.855	0.00042795
Fructose	12.839	1.03E-10	D-Pinitol	3.8213	0.00046359
Pyruvic acid	12.746	1.15E-10	Ethanolamine	3.6578	0.00068602
D-lyxose	12.729	1.18E-10	Levoglucosan	3.6411	0.00071415
Quinic acid	12.606	1.37E-10	4-Aminobutanoic acid	3.5898	0.00080864
Cellobiose	12.438	1.69E-10	Galactitol	3.5469	0.00089736
D-malic acid	11.731	4.19E-10	Alpha ketoglutaric acid	3.4181	0.001229
Glyceric acid	11.589	5.05E-10	Hydroxyproline	3.418	0.0012296
Glycine	11.393	6.54E-10	Scyllo-Inositol	3.3429	0.001479
Uracil	10.986	1.14E-09	Palatinitol	3.3327	0.0015167
Glycerol	10.717	1.65E-09	Syringic acid	3.2911	0.0016813
Unknown 1	10.383	2.64E-09	Unknown 3	3.2501	0.001861
D-glucose	10.045	4.29E-09	L-methionine	3.2077	0.0020682
D-mannose	9.4276	1.08E-08	Unknown 2	3.1477	0.0024024
DL-isoleucine	9.1292	1.70E-08	D-sorbitol	3.1378	0.0024626
Maltose	8.9725	2.17E-08	Oxalic acid	3.1243	0.002547
Myo-Inositol	8.5238	4.44E-08	Fumaric acid	2.9608	0.0038437
Erythritol	8.4978	4.63E-08	Allantoin	2.8219	0.0054677
Phosphoric acid	8.086	9.13E-08	Citramalic acid	2.8096	0.0056413
L-valine	7.9591	1.13E-07	Indole-3-acetic acid	2.6784	0.0078888
Unkown 7	7.5592	2.25E-07	Hydroxycinnamic acid	2.6477	0.0085349
L-Threonic acid	7.4433	2.75E-07	Gluconic acid	2.5823	0.010096
Raffinose	7.4075	2.93E-07	L-ornithine	2.5793	0.010174
Succinic acid	7.3563	3.21E-07	Unknown 6	2.5619	0.010641
Cinnamic acid	6.7692	9.30E-07	L-glutamic acid	2.5032	0.012376
Tyramine	6.7556	9.54E-07	Aspartic acid	2.3588	0.017963
Galactinol	6.6665	1.13E-06	L-ascorbic acid	2.2068	0.026595
Malonic acid	6.0554	3.64E-06	Glycolic acid	2.1566	0.030268

Glucopyranose	6.0388	3.76E-06	Unknown 4	2.0356	0.041326
Mucic acid	5.9075	4.88E-06			
Glucose-6-phosphate	5.8704	5.26E-06			
L-proline	5.8245	5.77E-06			
Melezitose	5.6703	7.87E-06			
Tyrosine	5.5995	9.10E-06			
Putrescine	5.0967	2.60E-05			
Galacturonic acid	5.0068	3.16E-05			
L-alanine	4.9179	3.83E-05			
Citrulline	4.7434	5.60E-05			

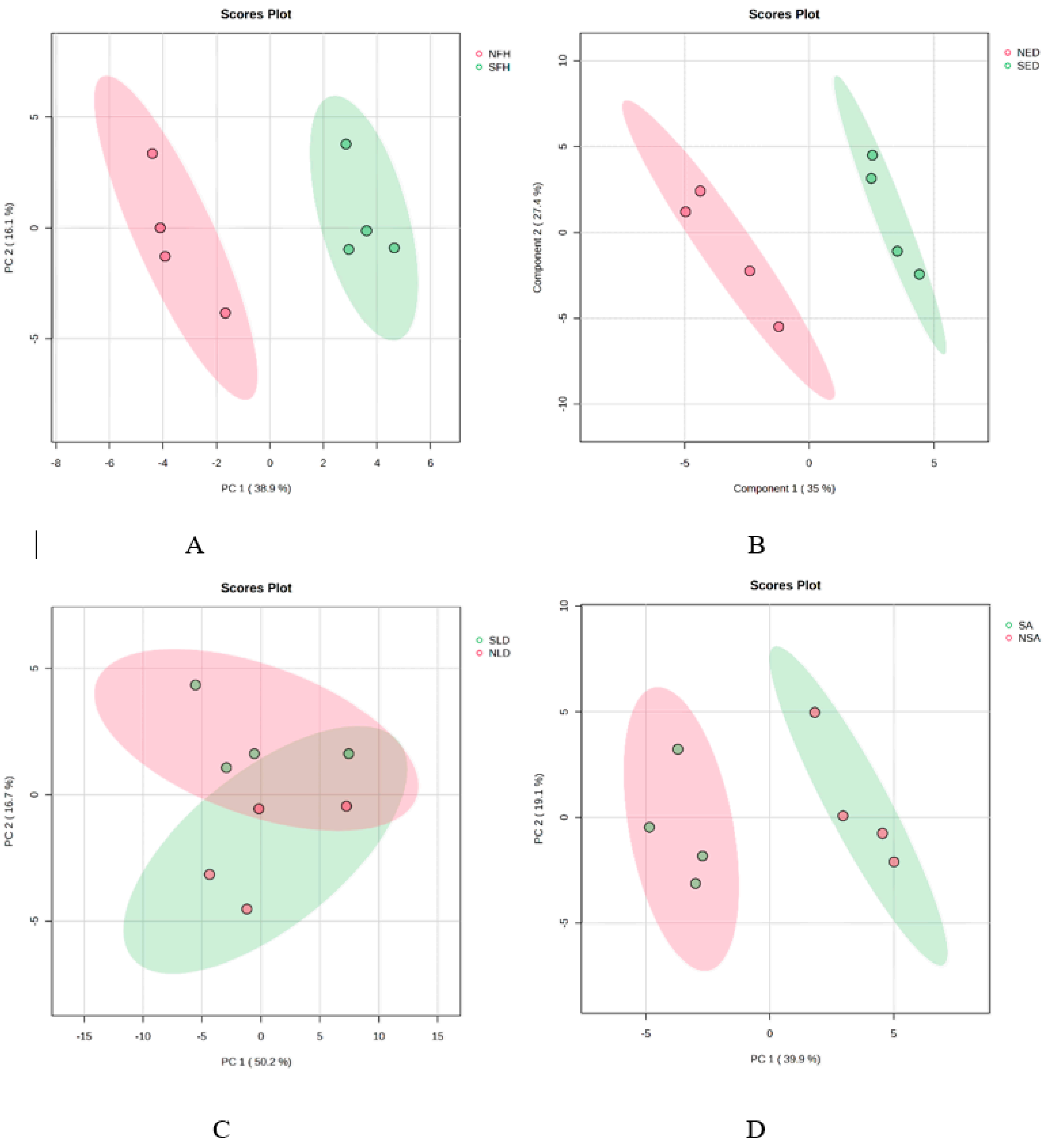

NFH - Non-senescent fully hydrated (> 85% RWC) NED - Non-senescent early dehydration (75-60% RWC)
SFH - Senescent fully hydrated (> 85% RWC) SED - Senescent early dehydration (75-60% RWC)
NSA - Non-senescent air-dry (<5% RWC) NLD - Non-senescent early dehydration (55-40% RWC)
SA - Senescent air-dry (<5% RWC) SLD - Senescent early dehydration (55-40% RWC)

Figure S1: Score plots of *E. nindensis* dehydration stages comparing the differences in NST and ST based on principal component analysis. (A) separation between fully hydrated ST and NST, (B) separation between early dehydrated ST and NST, (C) separation between late dehydrated ST and NST and (D) separation between air dry ST and NST. Samples were classified based on RWCs, which are depicted in various colours.

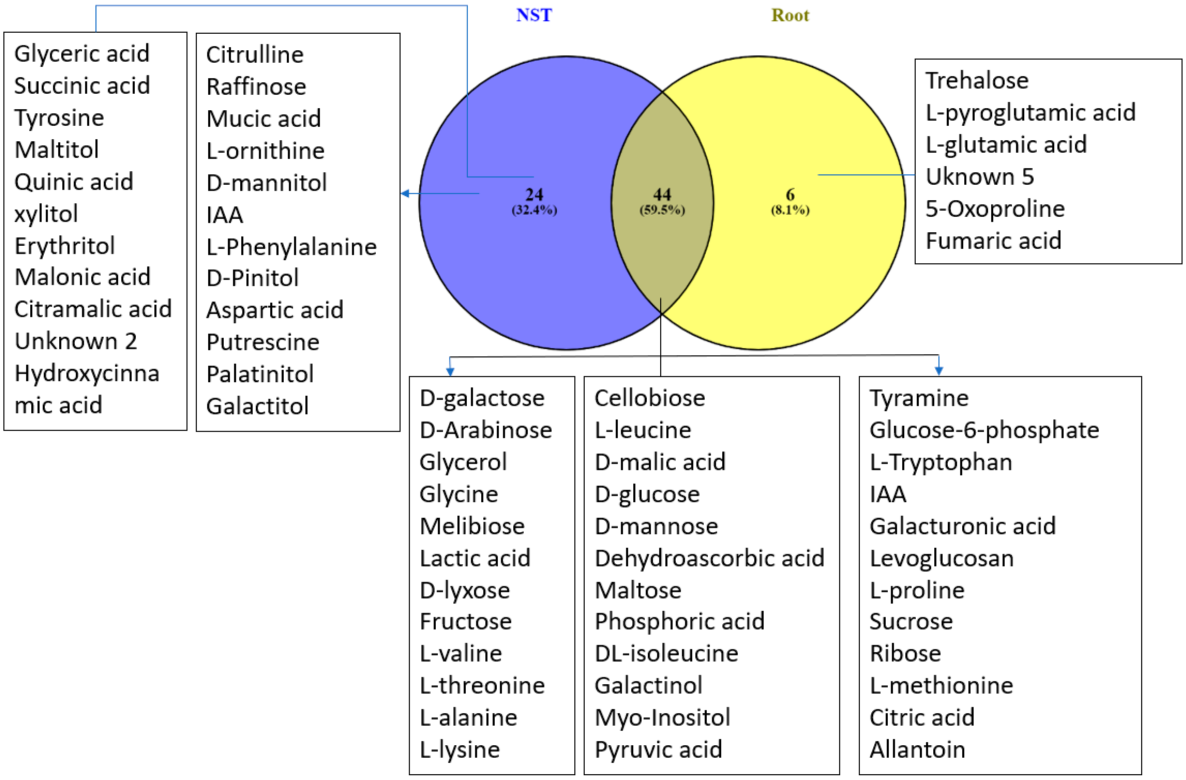

Figure S2: Venn diagrams of differential significant metabolites among non-senescent tissue and roots, metabolites exclusive to each tissue type, and those shared among the two tissue types. Statistical significance was carried out with a p-value < 0.05.
